# An iPS‐derived in vitro model of human atrial conduction

**DOI:** 10.14814/phy2.15407

**Published:** 2022-09-18

**Authors:** Sherri M. Biendarra‐Tiegs, Sergey Yechikov, Bhupinder Shergill, Brittany Brumback, Kentaro Takahashi, Venktesh S. Shirure, Ruth Estelle Gonzalez, Laura Houshmand, Denise Zhong, Kuo‐Chan Weng, Jon Silva, Timothy W. Smith, Stacey L. Rentschler, Steven C. George

**Affiliations:** ^1^ Department of Biomedical Engineering University of California, Davis Davis California USA; ^2^ Department of Biomedical Engineering Washington University in St. Louis St. Louis Missouri USA; ^3^ Department of Medicine Washington University in St. Louis St. Louis Missouri USA; ^4^ Department of Developmental Biology Washington University in St. Louis St. Louis Missouri USA

**Keywords:** atrial fibrillation, conduction velocity, ex vivo, in vivo, refractory period, wavelength

## Abstract

Atrial fibrillation (AF) is the most common arrhythmia in the United States, affecting approximately 1 in 10 adults, and its prevalence is expected to rise as the population ages. Treatment options for AF are limited; moreover, the development of new treatments is hindered by limited (1) knowledge regarding human atrial electrophysiological endpoints (e.g., conduction velocity [CV]) and (2) accurate experimental models. Here, we measured the CV and refractory period, and subsequently calculated the conduction wavelength, in vivo (four subjects with AF and four controls), and ex vivo (atrial slices from human hearts). Then, we created an in vitro model of human atrial conduction using induced pluripotent stem (iPS) cells. This model consisted of iPS‐derived human atrial cardiomyocytes plated onto a micropatterned linear 1D spiral design of Matrigel. The CV (34–41 cm/s) of the in vitro model was nearly five times faster than 2D controls (7–9 cm/s) and similar to in vivo (40–64 cm/s) and ex vivo (28–51 cm/s) measurements. Our iPS‐derived in vitro model recapitulates key features of in vivo atrial conduction and may be a useful methodology to enhance our understanding of AF and model patient‐specific disease.

## Graphical abstract

We present an induced pluripotent stem‐derived in vitro model of human atrial conduction. The design includes a linear 1D spiral design that significantly increases conduction velocity and thus recapitulates key features of in vivo atrial conduction. The model may be a useful methodology to enhance our understanding of atrial fibrillation and model patient‐specific disease.

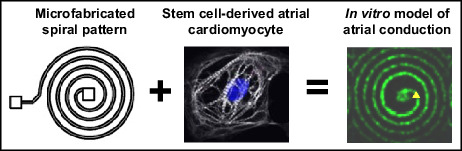

## 1 INTRODUCTION

Atrial fibrillation (AF) represents an increasingly common heart rhythm disorder, afflicting nearly 1 in 10 adults over age 65 in the United States (January et al., 2014; Mwozaffarian et al., 2015). The total healthcare burden is predicted to increase as the population ages, with prevalence in the United States estimated to grow from approximately 5.2 million in 2010 to 12.1 million in 2030 (Colilla et al., 2013). However, despite increased mortality and morbidity risks associated with AF (Lip et al., 2016), treatment options remain limited for these patients. Current therapies include pharmaceutical and surgical interventions, both of which are only effective in a subset of patients and have the potential to increase the risk of arrhythmia (Staerk et al., 2017). One of the major challenges is the heterogeneous nature of the patient population, whose disease may be caused and propagated by a range of underlying mechanisms (Nattel et al., 2020). For that reason, a more complete understanding of the pathophysiology of AF will improve disease management.

A variety of experimental disease models have been developed to better understand the mechanisms underlying AF (van Gorp et al., 2020). Human‐induced pluripotent stem cell‐derived cardiomyocytes (hiPSC‐CMs) have gained traction in recent years as the generation of atrial‐like cardiomyocytes (hiPSC‐aCM) has become feasible (Benzoni et al., 2020; Goldfracht et al., 2020; Marczenke et al., 2017; Nakanishi et al., 2019). Advantages of hiPSC‐aCMs include their renewable nature and human origin, the latter of which can potentially avoid electrophysiological differences between humans and animal models (van Gorp et al., 2020). Furthermore, these cells can be generated from AF patients providing the potential for replicating patient‐specific diseases, or be genetically modified for the mechanistic study of genetic, environmental, and/or structural contributors to disease.

Thus far, numerous mechanistic concepts to explain the initiation and maintenance of AF have been proposed, including several reentrant mechanisms. Reentry refers to the repeated activation of the cardiac tissue by a persistent excitation wave front and thus can result in the propagation of abnormal electrical activity within the heart. Previously, hiPSC‐CMs have been used to model spontaneous reentry phenomena in tissue rings (Goldfracht et al., 2020; Zhang et al., 2020). Conditions that support reentry include reduced conduction velocity (CV) (allowing previously refractory cardiac tissue to repolarize and become excitable) or reduced effective refractory period (ERP) that is generally manifested by a shorter action potential duration (APD). Since both a reduced CV and a reduced ERP enhances the susceptibility of cardiac tissue for reentry, a convenient index that captures both is the product of the two or the conduction wavelength (WL). Shorter WLs encourage reentry circuits to form within the same atria, thus increasing the risk of sustained AF (Waks & Josephson, 2014); as such, WL may be a more reliable predictor of arrhythmia than a CV or ERP/APD alone (Rensma et al., 1988). Accordingly, previous models utilizing stem cell‐derived atrial cardiomyocytes have examined CV and APD/ERP as indicators of enhanced AF risk (Goldfracht et al., 2020; Laksman et al., 2017); however, a major limitation of these models has been relatively (compared with in vivo [Fukumoto et al., 2016; Kistler et al., 2004; Kojodjojo et al., 2006; Weber et al., 2011]) slow CV (<6 cm/s). Furthermore, there is limited information characterizing electrophysiological endpoints of the human atria in vivo in AF.

In this study, we first made in vivo (normal controls and patients with AF) and ex vivo measurements of CV and ERP in adult human hearts to provide a physiologically relevant benchmark. We then utilized microfabrication technology to create a model system of human atrial conduction using hiPSC‐aCMs. The model employs a linear spiral design that enables one to capture the entire depolarization and repolarization event in the field of view of a standard epifluorescent microscope and thus provides a direct visual measurement of WL (as well as CV and APD/ERP such that WL can also be calculated). The model produces rapid CV (34–41 cm/s) that mimics the in vivo (40–64 cm/s) and ex vivo (28–51 cm/s) measurements. As such, our in vitro induced pluripotent stem (iPS)‐derived model of atrial conduction more accurately reflects human atrial electrophysiology compared with 2D monolayers and may be useful in patient‐specific modeling of AF.

## 2 METHODS

### 2.1 In vivo electrophysiological recordings of the human atria

All procedures associated with the in vivo electrophysiological recordings were approved by the Institutional Review Board at Washington University in St. Louis, and written informed consent was obtained from each subject. We enrolled a total of eight patients for in vivo electrophysiological recording of the atria (Table 1). Patients undergoing electrophysiology study and ablation for idiopathic ventricular tachycardia, idiopathic premature ventricular contractions, or atrioventricular nodal reentrant tachycardia were considered controls. The control patients had no evidence of structural heart disease (by echocardiography), no history of AF or other atrial arrhythmias, and were in normal sinus rhythm on the day of the procedure. AF patients were those scheduled for an ablation procedure (radiofrequency or cryoenergy) for paroxysmal AF. AF patients also demonstrated no evidence of structural heart disease (by echocardiography), no clear underlying causes of AF (e.g., thyrotoxicosis, pulmonary embolism, history of cardiac surgery), and were in normal sinus rhythm on the day of the procedure.

**TABLE 1:** Human subjects for in vivo measurements

Sample ID	Age	Sex	Category	Diagnosis
1	65	F	Control	AVNRT
3	45	M	Control	PVC
7	75	M	Control	PVC
8	50	F	Control	AVNRT
2	58	M	AF	Paroxysmal AF
4	51	M	AF	Paroxysmal AF
5	75	F	AF	Paroxysmal AF
6	41	M	AF	Paroxysmal AF

Abbreviations: AF, atrial fibrillation; AVNRT, atrioventricular nodal reentrant tachycardia; PVC, premature ventricular contractions.

For the electrophysiology procedure, venous access was obtained in the right and/or left femoral veins and hemostatic sheaths were placed. As part of the clinically indicated procedure, a deflectable decapolar catheter (Daig Inquiry) was used to record electrograms from the coronary sinus. The decapolar catheter had 2‐5‐2 mm spacing, yielding five bipoles arrayed curvilinearly over 20 mm. For the study procedure, the decapolar catheter was first placed along the lateral right atrial wall. After the study, pacing procedures were performed in the right atrium, the decapolar catheter was repositioned to the coronary sinus to perform the left atrial (LA) portion of the pacing/recording. In each position, burst pacing was performed to assess CV, and atrial extrastimuli were used to assess atrial ERP. Pacing outputs were 10 V and 2 ms. The pacing was performed from either the proximal or distal bipole at three cycle lengths: 400, 500, and 600 ms. The conduction time was measured over the 20 mm length of the decapolar catheter. Dividing 20 mm by the conduction time yielded the CV. Atrial extrastimuli were also applied with pacing drive cycle lengths of 400, 500, and 600 ms. The extrastimulus was decremented with each successive drive until the atrial ERP was reached. The in vivo data were all collected prior to any other electrophysiology study or ablation.

### 2.2 Ex vivo electrophysiological recordings of human atrial tissue slice

Nonfailing human hearts were obtained from Mid‐America Transplant Services (MTS) in St. Louis, MO. Experimental protocols were approved by the Washington University in St. Louis Institutional Review Board (IRB). Informed consent was obtained for all tissue used in this study. Methods described in this section were performed in accordance with all human research guidelines. Prior to organ harvest, intact hearts were perfused and arrested with a cold cardioplegic solution (Belzer UW® Cold Storage Solution, Bridge to Life Ltd.). After procurement, hearts were immediately submerged in cold cardioplegia and transported on ice. The patient characteristics of these donors are summarized in Table 2.

**TABLE 2:** Human subjects for ex vivo atrial slice samples

Sample ID	Age	Sex	BMI	Hypertension	Diabetes	Smoking	AF	CAD	Heart Operation
H91	46	M	28.56	Y	Y	Y	N	N	N
H98	30	M	22.46	N	N	Y	N	N	N

Abbreviations: AF, atrial fibrillation; BMI, body mass index; CAD, computer‐aided design.

LA tissue was obtained from the posterior region of the LA between the four pulmonary veins (LA “box”). Right atrial (RA) tissue was obtained from the crista terminalis below the sinoatrial node region (Figure S1a,b). Atrial tissue was cut into a 1 × 1 cm block and glued to 4% agarose gel (Figure S1c) on the tissue holder of a high‐precision vibrating microtome (7000 smz‐2, Campden Instruments Ltd.). The slicing chamber was filled with cold (4°C) oxygenated (95% O_2_, 5% CO_2_) modified Krebs‐Heinseleit solution containing the excitation–contraction uncoupler 2,3‐butanedione (BDM) (in mM: NaCl 128.2; KCl 4.0, glucose 10; NaHCO_3_ 20; NaH_2_PO_4_ 1.19; taurine 10; BDM 30; MgCl_2_ 1; pH 7.4). The outer chamber was constantly refilled with ice to maintain 4°C in the slicing chamber. The microtome was set to 400 μm cutting thickness, 0.02 mm/s advance speed, 2 mm horizontal vibration amplitude, and 80 Hz vibration frequency. The microtome's *z*‐axis vibration was also calibrated prior to each experiment with the ceramic blade to <0.5 μm, which limited damage to cardiomyocytes during the slicing procedure. After each slice was cut, it was transferred to oxygenated (95% O_2_, 5% CO_2_) Ca^2+^ reintroduction solution (in mM: NaCl 128.2; KCl 4.0, glucose 10; NaHCO_3_ 20; NaH_2_PO_4_ 1.19; taurine 10; BDM 30; MgCl_2_ 1, CaCl_2_ 0.9; pH 7.4) and incubated for 30 min. After incubation in the reintroduction solution, slices were maintained in the Krebs‐Heinseleit washing solution (in mM: NaCl 128.2; KCl 4.0, glucose 10; NaHCO_3_ 20; NaH_2_PO_4_ 1.19; MgCl_2_ 1, CaCl_2_ 1.8; pH 7.4) for 20 min to wash out BDM and gradually rewarm to 37°C.

Atrial slice electrophysiology was studied with the optical mapping technique as previously reported (Kang et al., 2016). All optical mapping studies were performed at 37°C in the same washing solution described above. Mapping data were acquired using an Ultima‐L CMOS camera system (SciMedia). Transmembrane potential (*V*_m_) was measured with di‐4‐ANEPPS (Life Technologies). The dye was loaded very slowly on top of the slice and incubated for up to 30 min. The excitation–contraction uncoupler, blebbistatin (0.2 mg/mL, Cayman Chemical), was loaded in a similar manner to decrease noise from motion artifacts. A 50 mm Nikon lens was used to project a 1 × 1 cm field to a 100 × 100 pixel CMOS sensor to measure optical action potentials.

All slices were paced with a custom‐designed platinum electrode (Figure S1d). The pacing stimulus was set at 1.5 times the amplitude of the pacing threshold and 2 ms pulse duration. Action potentials were recorded at a cycle length 1000 ms. For extrastimuli, a pacing drive train of 10 S1 at a cycle length of 1000 ms was used, followed by an S2 with a 600 ms coupling interval, which was reduced in 10 ms intervals until loss of capture to determine the ERP.

We excluded the slices in which the fluorescent signal was recorded in <50% of the entire slice. A custom data analysis program, Rhythm, developed by the Efimov laboratory for optical mapping data, was used to analyze *V*_m_ (Laughner et al., 2012). Action potential optical signals were low‐pass filtered at 100–150 Hz, spatially averaged at 7 × 7 pixels, normalized from 0 to 1, and fluorescent drift was removed with a first‐order fitted curve, if needed. The activation map was plotted by calculating activation time points at 50% of action potential upstroke amplitude. APD was measured at 4–5 points, where the signal did not have the motion artifact. The motion artifact was defined as (1) the action potential trace going below baseline or (2) derivative of the action potential APD90 calculated at 90% repolarization/relaxation, respectively. CV was calculated using a semiautomated Matlab function ORCA (Doshi et al., 2015). Longitudinal and transverse CV were calculated in a specific direction, where the maximum and minimum CV were measured, respectively.

### 2.3 hiPSC culture and differentiation to atrial cardiomyocytes

hiPSCs (C2A [Sepac et al., 2012] and 6‐9‐9 [Yu et al., 2009]) were cultured in mTeSR1 (Stem Cell Technologies #85850) on growth‐factor reduced Matrigel (Corning # 354230) and passaged using ReLeSR (Stem Cell Technologies #05873). hiPSCs were replated in 12‐well plates at 80% density using Accutase dissociation reagent (ThermoFisher # A1110501) 1 day before initiating differentiation.

On day 0 of differentiation, media were changed to RPMI‐1640 with l‐glutamine (ThermoFisher # 11879020) containing B‐27 supplement without insulin (ThermoFisher # A1895602), and 100% confluent hiPSCs were treated with 12 μM glycogen synthase kinase (GSK)3 inhibitor—CHIR99021 (LC Laboratories #C‐6556) for 24 h. On day 3, cells were treated with 5 mM Wnt inhibitor, IWP‐2 (Tocris # 3533) and 1 μM retinoic acid (RA) (Sigma # R2625) for 48 h (Lian et al., 2012, 2013; Zhang et al., 2011). After day 7, cells were maintained in RPMI‐1640 with l‐glutamine and B‐27 supplement with insulin (ThermoFisher # 17504‐001) and maintained until day 60 to achieve a highly enriched population of hiPSC‐aCM.

### 2.4 Patch‐clamp recording of action potentials

Action potentials of single hiPSC‐derived cardiomyocytes were recorded at 37°C using Axon Axopatch 200B amplifier interfaced with digitizer Digidata 1440A controlled by pClamp software (Molecular Devices). The external recording solution was Tyrode's solution, composed of 140 mM NaCl, 5 mM KCl, 1 mM CaCl_2_, 1 mM MgCl_2_, 10 mM HEPES, and 10 mM glucose; pH was adjusted to 7.4 using NaOH. The pipette solution contained 120 mM K‐glutamate, 25 mM KCl, 1 mM CaCl_2_, 1 mM MgCl_2_, and 10 mM HEPES; pH was adjusted to 7.4 using KOH. The resistance of borosilicate pipettes ranged between 3 and 5 MΩ. Signal was processed with a 5 kHz low‐pass filter and action potential parameters were measured in pClamp.

### 2.5 2D microelectrode array recordings

At least 5 days before recording, 3.5 × 10^5^ cell/ml hiPSC‐aCM were seeded onto the Matrigel‐coated electrode area of the probe (Alpha Med Scientific, MED‐P545A) to form a monolayer. The recording was done in Tyrode's solution with stimulation from microarray electrode 1 (2 Hz, 5 ms square pulses with output current not exceeding 100 μA) on an MED64 MEA system (Alpha Med Scientific). Horizontal, vertical, and diagonal vectors from the conduction map were averaged to report the CV of the monolayer.

### 2.6 Microfabrication of linear spiral device design

To enhance CV and visualize the WL, we created a spiral design for the hiPSC‐aCMs. In this approach, a long narrow line of cells shaped into a spiral minimizes the dimensions utilized to observe the depolarization and repolarization of the cardiomyocytes. To create this design, we created the spiral shape using standard computer‐aided design software (AutoCAD) and then used standard methods of soft lithography to create a soft mold of polydimethylsiloxane. The PDMS mold was then reversibly attached to a glass slide; Matrigel™ (Corning #354230) was injected into the spiral channel to create a thin coat on the glass surface in the shape of the mold; the PDMS mold was peeled off; the glass slide coated with pluronic F‐68 to minimize nonspecific cell attachment; the slide was washed, and the hiPSC‐aCMs were coated over the surface at a concentration of 2.5 × 10^5^ cells/ml. The cells were allowed to attach overnight, and unattached cells were removed. The attached cells were maintained in RPMI‐1640 with l‐glutamine and B‐27 supplement with insulin (ThermoFisher # 17504‐001) for 5–9 days prior to electrophysiological recordings. To maximize experimental replicates, our design included five spirals on each glass slide. We etched gold‐plated glass slides to create electrodes (Weng et al., 2020) as previously described such that each spiral of hiPSC‐aCMs could be electrically paced.

### 2.7 Spiral device recordings and processing

Prior to recording, optical action potential (Fluoview confocal microscope [Olympus]), cells were incubated for 30 min at 37°C with Fluvolt (ThermoFisher #F10488) voltage‐sensitive dye and then washed twice with Tyrode's solution. Recordings were performed at 30 fps using either a 1.25X or 2X PlanApo N objective (Olympus) in order to fit the entire spiral design into the field of view. Cells were stimulated with 5–15 mV square pulses at 2 Hz (IonOptix Myopacer) and images were collected. The line scan resonant mode (150 fps) was utilized to measure APD and travel time through the spiral device. Temporal Fluovolt fluorescence profiles from the same line scan recording were extracted from the region at the electrode and at the terminal of the spiral and smoothed using the Markov chain method. Then, APD was measured at 80% of maximal amplitude. Travel time was calculated as the difference between action potential peak times in each region. The Fluovolt fluorescence intensity profile along a 10 pixels‐wide line following the cardiomyocyte path in a spiral device was extracted for every time frame (30 ms) using ImageJ software. Each recording typically contained 300 frames (10 s). All subsequent processing was done in R. Signal for each frame was filtered with a 0.005 low‐pass Butterworth filter; each profile data point was scaled to the corresponding average value calculated for 300 frames; then, the resulting signal was amplified to visualize conduction wave progression. For each recording, a minimum of 10 electrode area waves were measured at 80% of maximal wave amplitude and averaged.

### 2.8 qPCR

Cells were collected in Trizol reagent (Thermo Fisher #15596026) and stored at −80°C. Following phenol–chloroform extraction, RNA from the aqueous phase was isolated with an RNeasy kit (Qiagen #74106), purified from genomic DNA contaminants using a gDNA cleanup kit (Thermo Fisher #K2981), and transcribed into cDNA with a high‐capacity cDNA reverse transcription kit (Thermo Fisher #4368814). qPCR reactions were assembled with PowerUp Sybr Green mastermix (Thermo Fisher #A25776) according to the manufacturer's instructions (total volume: 10 μl) and performed in 384‐well plates on a ViiA7 real‐time PCR system (Thermo Fisher). Primer sequences are provided in Table S1 (10.6084/m9.figshare.19285397). Relative gene expression was calculated using GAPDH as a housekeeping gene.

### 2.9 Immunofluorescence staining

Cells grown on a glass coverslip were (1) fixed for 15 min at room temperature in 4% paraformaldehyde/PBS solution at pH 7.4, (2) postfixed in acetone for 5 min, boiled for 10 min in pH 6 citrate buffer, and (3) blocked with 1% bovine serum albumin, 4% goat serum, and 0.05% Tween‐20. Then, the slides were incubated overnight at 4°C with antibodies against the cardiac marker Troponin T (Developmental Study Hybridoma Bank, CT3), the atrial‐specific isoform of myosin light chain Mlc2a (Synaptic Systems #310011), and the ventricular‐specific isoform of myosin light chain Mlc2v (Synaptic Systems #310203). Nuclei were counterstained with DAPI.

### 2.10 Statistical analysis

Statistical analysis was performed using GraphPad Prism 8 or R. *T*‐tests (paired or unpaired) were performed where indicated. Analysis of variance (ANOVA) followed by a Tukey's test for post hoc comparisons was performed as indicated. All data are represented as the mean with the standard error of the mean included.

## 3 RESULTS

### 3.1 In vivo and ex vivo benchmarks

**FIGURE 1:**
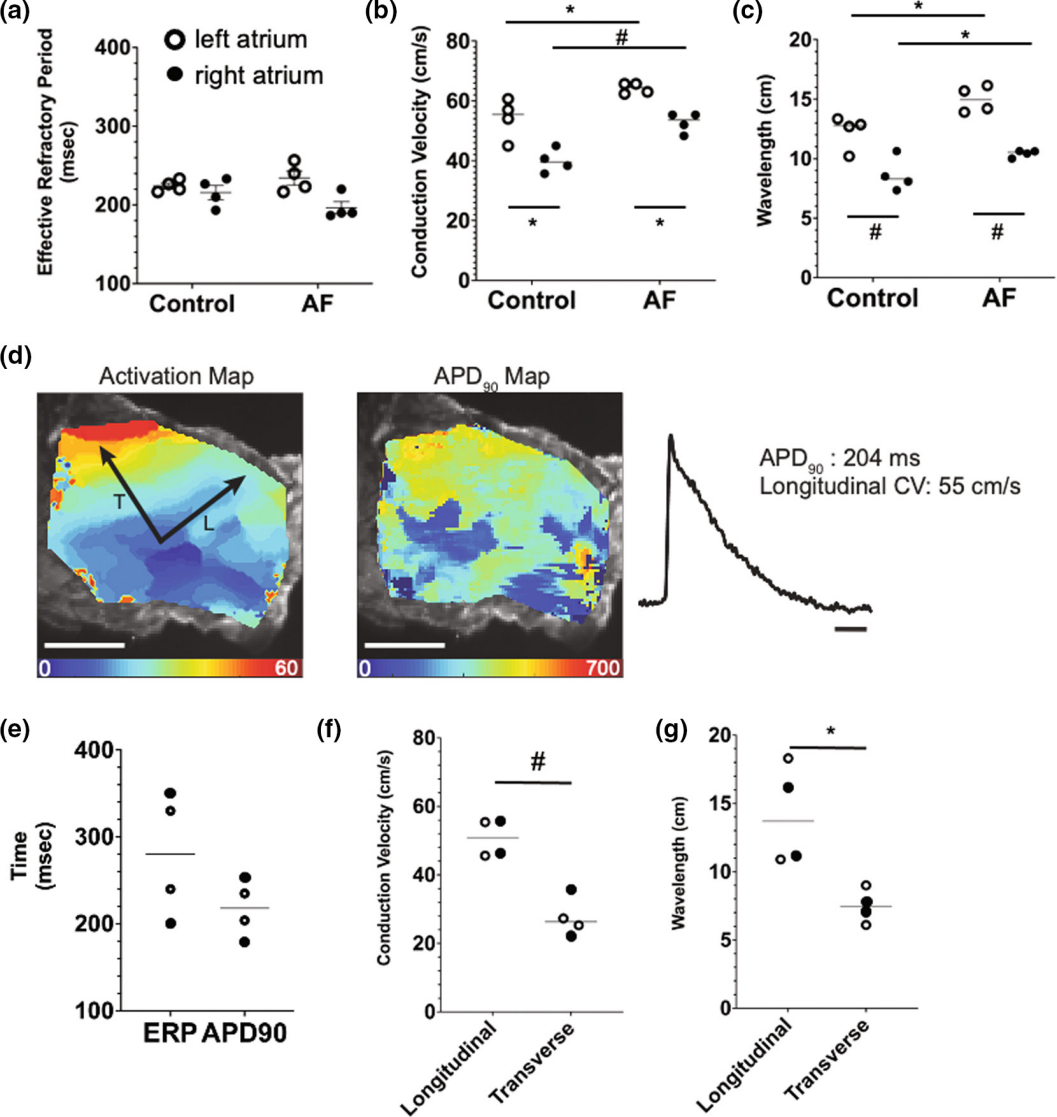
In vivo and ex vivo electrophysiological endpoints of human atria. (a) Effective refractory period (ERP), (b) conduction velocity (CV), (c) wavelength in the left atrium (LA) and right atrium of control and atrial fibrillation human subjects in vivo, (d) from the ex vivo human prep, representative activation map (left), APD map demonstrating APD_90_ (middle), and action potential trace (right) obtained from LA slice at baseline conditions. CV was measured in the transverse (T) and longitudinal (L) directions indicated by black arrows in the activation map. Measured APD_90_ and L–CV were 204 ms and 55 cm/s, respectively, and (e–g) from the ex vivo human prep, comparison of electrophysiological parameters for (e) the action potential duration at 90% repolarization (APD_90_) and the ERP, (f) longitudinal and transverse CV, and (g) longitudinal and transverse wavelength. *p < 0.05; ^#^ *p* < 0.01.

To create accurate and appropriate benchmarks for our in vitro model, we measured electrophysiological endpoints of human atria both in vivo and ex vivo. We measured CV and ERP (and calculated the WL as the product of CV and ERP) in eight human subjects, four controls without AF and four patients with paroxysmal AF, who were undergoing elective ablation (Table 1). There was no difference in the age of the subject between control (57 ± 7.6 years) and AF (56 ± 8.3 years). The measurements were performed in both the LA and RA, and at three different drive cycle lengths (400, 500, and 600 ms). There was no dependence of ERP, CV, or WL on the drive cycle length (Figure S3). The ERP was not different between the left and right atria (paired *t*‐test), nor among the control and AF subjects (unpaired *t*‐test) (224 ± 7.4, 234 ± 18, 216 ± 18, 197 ± 16 ms, respectively) (Figure 1a). In contrast, CV and WL were both statistically higher in AF compared with control (unpaired *t*‐test) in both the LA and the RA as well as in the LA compared with the RA (paired *t*‐test) (54 ± 7, 40 ± 4, 64 ± 1.8, 53 ± 3.3 cm/s, respectively: 12 ± 1.4, 8.6 ± 1.4, 15 ± 1.1, 10 ± 0.3 cm, respectively) (Figure 1b,c).

In the ex vivo human atrial tissue slice prep, we were able to maintain viable tissue for 3 h. We measured the longitudinal and transverse CV, APD90, and ERP (and then calculated the WL as the product of CV and ERP) in two separate preps from two donors (Figure 1e–g). APD90 (218 ± 33 ms), ERP (280 ± 72 ms), longitudinal CV (51 ± 5.6 cm/s), transverse CV (28 ± 5.8 cm/s), longitudinal WL (14 ± 3.7 cm), and transverse WL (7.5 ± 1.3 cm) were all similar between the LA and the RA (Figure 1e–g). The longitudinal CV and WL were statistically higher (paired *t*‐test) compared with the transverse CV and WL (Figure 1e–g).

### 3.2 Human‐induced pluripotent stem cell‐derived atrial cardiomyocytes

**FIGURE 2:**
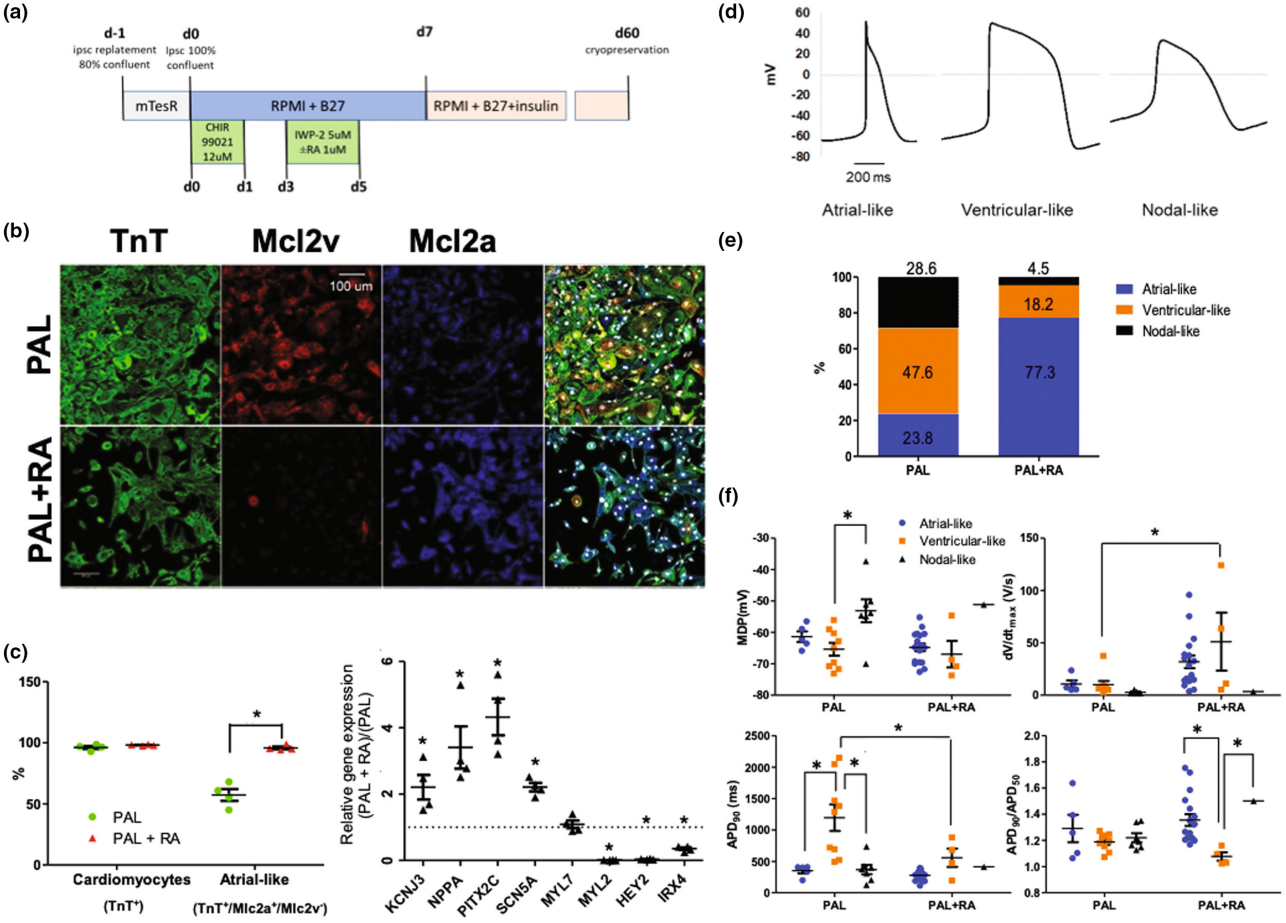
Generating iPS‐derived atrial cardiomyocytes. (a) Cardiomyocyte differentiation protocol outline, (b) immunofluorescent images of cardiomyocytes stained for ventricular‐ and atrial‐specific isoforms of myosin light chain, PAL—standard protocol, PAL + RA—atrial protocol, (c) characterization of cardiomyocyte populations based on fluorescent images analysis (cardiomyocytes positive for TnT, atrial—Mlc2a^+^/Mlc2v^−^) and qPCR, (d) characteristic path‐clamp traces of atrial‐like (MDP < −60 mV, APA > 100 mV, *dV*/*dt*_max_ >5 V/s, APD_90_ < 300 ms, APD_90_/APD_50_ > 1.2), ventricular‐like (MDP < −60 mV, APA > 100 mV, *dV*/*dt*_max_ >5 V/s, APD_90_ > 400 ms, APD_90_/APD_50_ < 1.2), and nodal‐like (MDP > −50 mV, APA < 100 mV, *dV*/*dt*_max_ <5 V/s) cardiomyocytes, (e) characterization of cardiomyocyte populations based on patch‐clamp data, and (f) electrophysiological parameters of iPSC‐derived cardiomyocytes measured by patch‐clamp. *p < 0.05. APD, action potential duration; MDP, minimal diastolic potential.

We efficiently differentiated two hiPSC lines (C2A and 6‐9‐9) into hiPSC‐aCM using a previously published protocol and the addition of RA (Lian et al., 2012, 2013; Zhang et al., 2011) (Figure 2a). In the absence of RA, immunofluorescence image analysis demonstrated that the differentiated cell population contained >90% troponin T‐positive cells (Figure 2b,c and Figure S4a). At day 60, we observed varying levels of Mlc2a expression in the hiPSC‐derived cardiomyocytes. We relied on the absence of Mlc2v expression to further classify cell populations. The addition of RA to the differentiation protocol (PAL+ RA) resulted in a dramatic increase in atrial‐like cells (TnT^+^/Mlc2a^+^/Mlc2v^−^—denoted hiPSC‐aCM) compared with the original Palecek protocol (Lian et al., 2013) (PAL) (95.8 ± 1.1% vs. 57.2 ± 4.8%) (unpaired *t*‐test). By qPCR, hiPSC‐aCM expressed higher levels of atrial‐specific transcripts (KCNJ3 221 ± 37%, NPPA 341 ± 63%, PITX2C 432 ± 110%) and lower levels of ventricular‐specific transcripts (MYL2 1.1 ± 0.2%, HEY2 3.7 ± 0.6%, IRX4 35 ± 3.9%) compared with the PAL protocol (100%) (ANOVA followed by Tukey's post hoc *t*‐test) (Figure 2c and Figure S4b).

We next performed patch‐clamp on the differentiated cells. The mixed hiPSC‐derived cardiomyocyte population had a minimal diastolic potential (MDP) of −60.4 ± 0.7 mV by patch‐clamp recordings. The differentiated cells exhibited classical nodal, atrial, and ventricular action potential profiles (Figure 2d) as well as profiles demonstrating characteristics of multiple cell types (e.g., ventricular and atrial). We classified the cells based on a depolarization (*dV*/*dT*_max_), repolarization speed (APD90/APD50), MDP, and action potential duration at 90% depolarization (APD90). Specifically, the subpopulation of cells with a maximal action potential amplitude (APD) < 100 mV, MDP > −50 mV, and a slow depolarization (*dV/dt*_max_ <5 V/s) was classified as nodal; and the subpopulation of cells with MDP < −55 mV and *dV/dt*_max_ >5 V/s was divided into ventricular cells (APD90 > 400 ms or APD90/APD50 < 1.2) and atrial cells (APD90 < 300 ms or APD90/APD50 > 1.2). The C2A cell line produced 77.3% cardiomyocytes with atrial‐like action potentials when differentiated by PAL + RA versus 23.8% when differentiated by PAL (Figure 2e), consistent with immunofluorescence and gene expression (Figure 2c). The hiPSC‐aCM had an MDP of −63.9 ± 1.3 mV, *dV*/*dt*_max_ of 27.1 ± 5.5 V/s, APD90 of 294.6 ± 24.3 ms, and APD90/APD50 of 1.34 ± 0.05. The addition of RA to the differentiation protocol did not significantly affect the electrophysiological parameters in hiPSC‐aCM; however, the ventricular‐like cardiomyocytes exhibited enhanced *dV*/*dt*_max_ (51.1 ± 3.5 vs. 10.1 ± 3.5 V/s) and shorter APD90 (557.3 ± 146.6 vs. 1194.9 ± 210 ms) (Figure 1f) (ANOVA followed by the post hoc *t*‐test).

### 3.3 2D depolarization of hiPSC‐aCM

**FIGURE 3:**
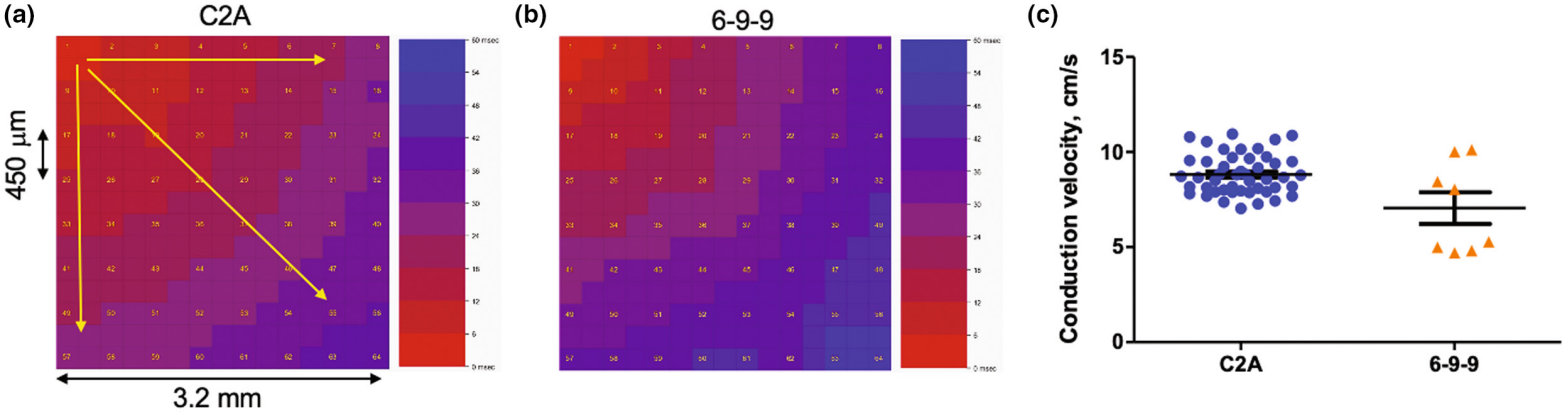
2D conduction velocity (CV). Monolayers of iPS‐aCM were plated on Med64 “probes” (8 × 8 microelectrode array) and CV was measured as a function of time postplating (days 5–9). There was no statistically significant change in CV over this time range. Representative depolarization maps for two different iPS lines: (a) C2A and (b) 6‐9‐9. Yellow arrows represent depolarization vectors for three measurements of conduction velocity on each array, (c) mean CV for two different iPS lines (mean ± SEM: 8.8 ± 0.15 and 7.0 ± 0.83 cm/s, respectively). Devices were stimulated from electrode 1 at 2 Hz to acquire the data.

Five days after plating cells on an MED64 probe, hiPSC‐aCM formed a contracting monolayer and were responsive to electrical stimulation, and 2 Hz stimulation was applied to override the internal pacing and minimize the effect of restitution. After five recovery days, we found no significant change in CV due to extended time (days 5–9) spent on the probe (data not shown). Depolarization waves are readily visible on the 2D MED64 MEA (Figure 3a,b). The mean CV, calculated by averaging the vertical, horizontal, and diagonal wave propagation vectors for the C2A and 6‐9‐9 cell lines (Figure 3a,b) was 8.8 ± 1 and 7.1 ± 2.4 cm/s, respectively (Figure 3c).

### 3.4 Linear 1D depolarization and repolarization in a microfabricated spiral design

**FIGURE 4:**
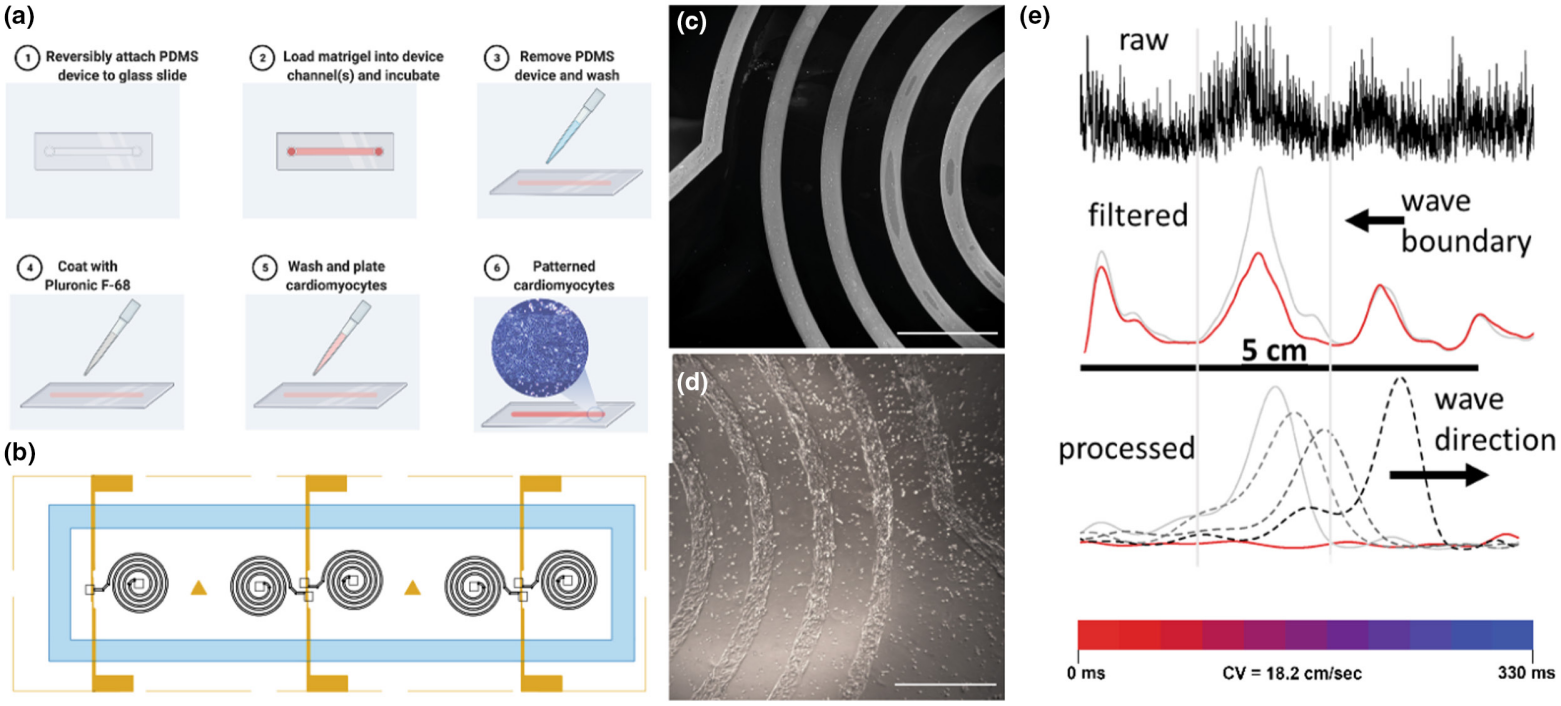
Spiral design, microfabrication, and data processing. (a) Methodology to create patterned Matrigel on glass slide. (b) Computer‐aided design drawing of the spiral design including the location of the pacing electrodes. Dimensions of each spiral are 5.5 cm in length, 200 μm in width, and 400 μm in spacing between each line. (c) Fluorescent image of laminin stain following Matrigel patterning (scale bar 1 cm). (d) Phase contrast image of iPSC‐aCM 7 days following seeding onto the spiral‐patterned Matrigel (scale bar 1 cm). (e) Representative tracing of fluorescent signal from iPSC‐aCM seeded on the spiral and labeled with fluovolt. The fluorescence intensity on the *y*‐axis is plotted against the length of the device on the *x*‐axis, before (“raw”) and after filtering (“filtered”); the red color represents no signal condition (baseline). An example of a detected conduction wave after subtracting baseline in four consecutive full frames taken at 33 fps (“processed,” red trace represents no signal condition, black arrow shows the direction of propagation).

Since the CV of the hiPSC‐aCM on the 2D MED64 MEA was significantly slower than our in vivo and ex vivo benchmarks, we sought an alternative design that would potentially enhance cell alignment and CV and also provide direct visualization of the WL (start of the repolarization to the end of depolarization). Constraining cardiomyocytes into narrow channels has been shown to enhance alignment (Chen et al., 2011; Mendoza et al., 2014; Wong et al., 2020); thus, we chose a narrow linear spiral design for our in vitro model of atrial conduction. The narrow line in a spiral shape would effectively model 1D atrial conduction in a geometry that encourages alignment while minimizing the physical dimensions in 2D such that the depolarization and repolarization events could be visualized using a standard epifluorescent microscope. The process for creating the spiral pattern of hiPSC‐aCM involved patterning a thin coat of Matrigel on a glass slide (Figure 4a). The glass slide also contained three pairs of gold electrodes positioned to pace either one or a pair of matching spirals (Figure 4b). The microfabrication process results in precise patterning of Matrigel as shown by laminin staining (Figure 4c).

To create our final design, we had two constraints: (1) a total linear length that exceeded the estimated WL from the 2D Med64 MEA (~2.4 cm [~8 cm/s × ~0.3 s] by at least two‐fold [to account for potential and desired higher CV]) and (2) a 2D footprint on the glass slide that could be visualized by a low magnification objective (1.25X or 2X) on an epifluorescent microscope (~65 mm^2^). We had two variables to adjust; namely, the width of the line (*d*_l_) and the width separating the lines (*d*_s_) as they form a spiral (Figure 4c). We examined a range of values for both *d*_l_ (200–400 μm) and *d*_s_ (200–600 μm) (Figure 2a,b). A smaller *d*_l_ and *d*_s_ are both advantageous as either minimizes the 2D footprint on the glass slide, thus facilitating a longer length of the spiral. We found that as *d*_s_ became smaller, there were more hiPSC‐aCM that formed connections (bridging) with adjacent lines (Figure 2c), and as *d*_l_ became smaller, there was a greater chance of having a break in the continuity of the hiPSC‐aCM along the patterned Matrigel (Figure 2d). Furthermore, continuity in the line of cells and bridging were also dependent on the cell‐seeding concentration (Figure 2e). The optimal values for *d*_l_ and *d*_s_ were found to be 200 and 400 μm, respectively, and the optimal cell‐seeding concentration was approximately 2 × 10^5^ cells/cm^2^ but may vary depending on the iPS parent cell (Figure 4c,d). This design resulted in a total linear length of 5.5 cm and a 2D footprint of 60 mm^2^, which could be visualized using a 2× objective. We also performed some experiments with an additional spiral turn producing a total length of 7.9 cm which could be visualized using a 1.25× objective.

**FIGURE 5:**
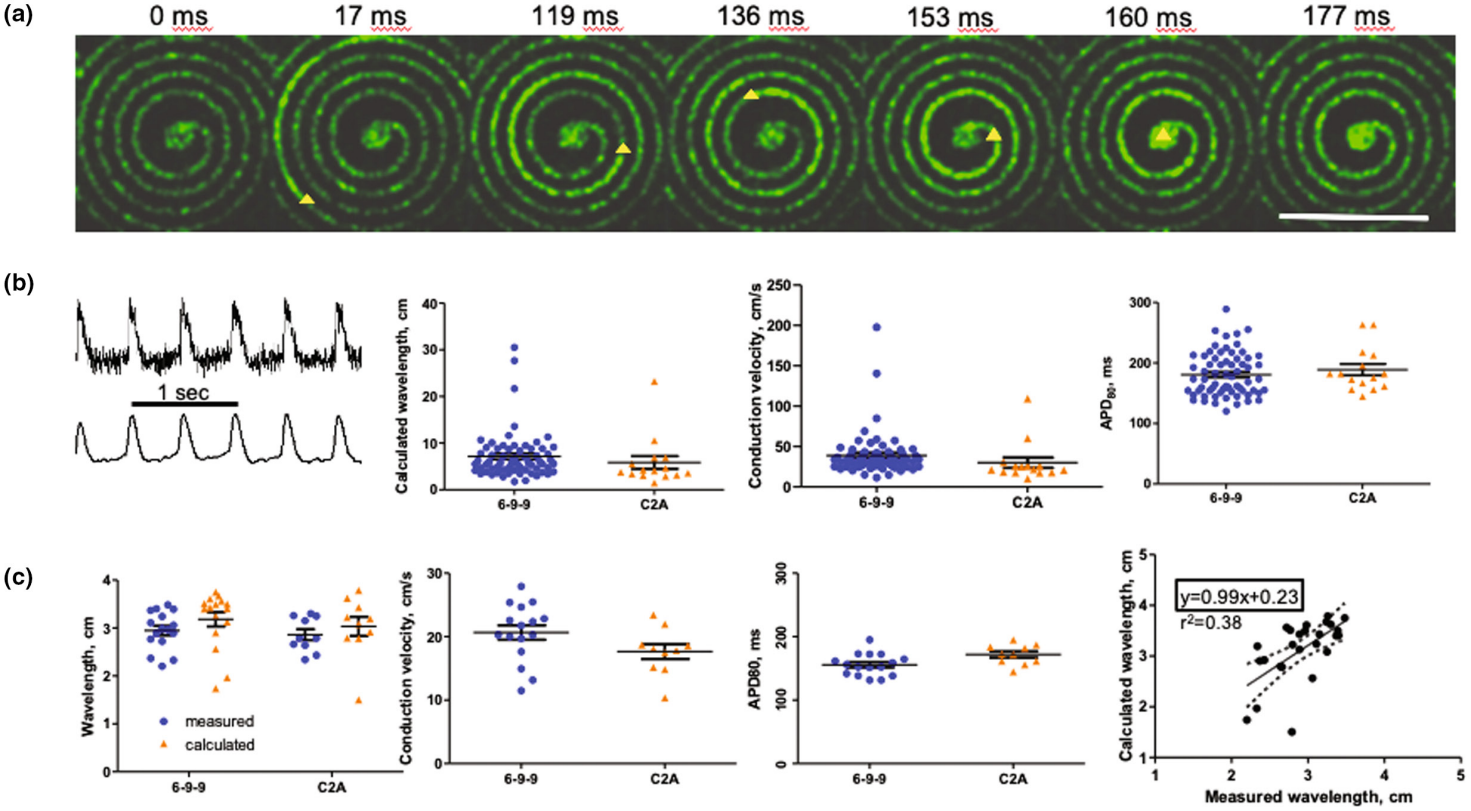
Electrophysiology of iPS‐derived cardiomyocytes on spiral‐patterned design. (a) Time‐lapse images of depolarization wave in a spiral (cell line C2A paced at 2 Hz; yellow triangle indicates the front of the wave; time represents the duration following stimulation from the electrode). Scale bar 5 mm. (b) Representative recordings acquired in line scan resonant mode from cardiomyocytes on the spiral device loaded with Fluovolt dye before and after filtering, conduction velocity, APD80, and calculated wavelength from two different iPS cell lines. (c) Conduction velocity, APD_80_, wavelength, and calculated wavelength from two different iPS cell lines from observations, where the wavelength is short enough to fit completely into a spiral design. The calculated and measured wavelength show a good correlation with a slope near 1.0. Devices were stimulated at 2 Hz during recording.

By 5 days after plating cells on the spiral device, hiPSC‐aCM formed a connected linear monolayer and propagated field potentials through the entire length of the spiral track. Between days 5 and 9, cells were loaded with the Fluovolt membrane voltage‐sensitive dye to visualize the conduction wave and stimulated at 2 Hz to override the internal pacing and minimize the effect of restitution. The entire spiral was optically recorded using a confocal microscope at 30 fps. After signal processing (see Methods), we could detect and visualize the conduction wave progression through the track of the device (Figures 4e and 5a, and Supplemental Video [10.6084/m9.figshare.19285376]). Additionally, the line crossing the stimulation electrode area and central region of the spiral were scanned at 150 fps in a line scan resonant mode to accurately measure the conduction time and the field potential duration.

We observed that the wave propagation time was lower than the APD for multiple spiral devices (in other words, the WL exceeded the linear length of the spiral), which made it impossible to visualize (and thus directly measured) the entire WL. We attributed this observation to an increase in the CV of the hiPSC‐aCM on the spiral device that exceeded expectations (two‐fold, based on design constraints). Indeed, we calculated conduction velocities of 41 ± 3.4 and 34 ± 6.4 cm/s accompanied by APD_80_ of 180.7 ± 4.5 and 188.9 ± 9.4 ms for the 6‐9‐9 and C2A cell lines, respectively (Figure 5b). These electrophysiological properties correspond to a calculated WL (product CV × APD_80_) of 7.2 ± 0.63 and 5.9 ± 1.4 cm (Figure 5b), which generally exceeded the linear length of the spiral design. Nonetheless, using the experimental conditions in which the WL was small enough to visualize (~50% of the total observations), we found a good correlation (*r* = 0.61 *p* < 0.001, slope of 0.99) between measured (directly observed) and calculated WLs (Figure 5c). This subset had CVs of 21 ± 1.1 and 18 ± 1.2 cm/s for the 6‐9‐9 and C2A cell lines, respectively, and an observed WL of <4 cm (Figure 5c).

## 4 DISCUSSION

AF remains a prevalent disease in the United States with limited treatment options. Significant interpatient heterogeneity in the underlying disease pathology contributes to the difficulty in disease management. iPS stem‐cell technology provides an opportunity to not only create models of human atrial conduction, but it also provides an opportunity for patient‐specific models. Current iPS‐derived models of human atrial conduction have been characterized by slow conduction (<6 cm/s) (Goldfracht et al., 2020; Laksman et al., 2017) relative to in vivo reports (Fukumoto et al., 2016; Kistler et al., 2004; Kojodjojo et al., 2006; Weber et al., 2011). Reproducing normal CV is an essential feature of modeling atrial conduction, as altered CV is thought to be a significant risk factor for AF. Our study presents a simple linear 1D model of human atrial conduction created from iPSC‐aCM. The model enhances CV by four‐ to five‐fold (34–41 cm/s), which recapitulates our in vivo (40–64 cm/s) and ex vivo (28–51 cm/s) measurements in human hearts. Our in vitro model may be a useful tool to better understand human atrial conduction and contribute to improved disease management strategies.

Our in vitro model achieved faster CV by constraining the geometry of the hiPSC‐aCMs into a relatively narrow line, consistent with previous studies that encourage alignment using narrow grooves (Chen et al., 2011; Mendoza et al., 2014; Wong et al., 2020) or patterned ECM (e.g., fibronectin) (Batalov et al., 2021). Interestingly, these previous studies utilized patterns ranging in width from 1 to 30 μm, whereas our patterned width of Matrigel was 200 μm. As such, it is not likely that our enhanced electrophysiological parameters can be explained solely by cell alignment. The enhanced CV may be due to long‐term self‐pacing stimulation augmented by the strictly vectorized field potential propagation granted by the narrow channel design. In vitro 3D models utilizing stem cell‐derived ventricular cardiomyocytes have produced a more rapid CV (15–50 cm/s) similar to our model using a variety of strategies including increasing the purity of the cardiomyocytes, manipulating the extracellular matrix, and using dynamic culture conditions (Herron et al., 2016; Jackman et al., 2016; Nunes et al., 2013; Ronaldson‐Bouchard et al., 2018; Zhang et al., 2013). These may also be effective strategies to enhance CV in hiPSC‐aCM. We also limited the overall length of our in vitro model to 7.9 cm, which allowed visualization of the entire spiral device using a 1.25× objective, but the lower magnification objective also compromises the signal‐to‐noise ratio. Hence, while longer spiral designs are possible and would enable the visualization of the entire WL over a wider range of conditions, we demonstrated a nearly 1:1 correlation between the measured (directly visualized) and calculated (product of CV × APD_80_) WL (Figure 5c).

Many previous studies have generated atrial‐like cardiomyocytes from induced pluripotent stem cells by either monolayer/GSK3 inhibition or embryoid bodies/TGFβ receptor activation approaches using RA to drive cardiac mesoderm toward atrial fate (Cyganek et al., 2018; Lee et al., 2017). In agreement with the previously published differentiation protocols, we observed expression of Mlc2a in all hiPSC‐derived CM, and the onset of Mlc2v expression was delayed by 20 days (on day 40) compared with reports on EB‐based differentiations. We found that reliable identification of a distinct mature atrial phenotype by either Mlc2a/Mlc2v protein expression or atrial‐specific marker gene expression required significant time in culture (~60 days). It is possible that other strategies may be able to create a more mature phenotype with less time in culture, such as conditioning (i.e., pacing), alternate extracellular matrices, co‐culture, soluble mediators, or surface chemistry/topography (Besser et al., 2018).

Our in vivo and ex vivo measurements of human atrial conduction are consistent with earlier in vivo reports of atrial CV (50–100 cm/s) and ERP (220–300 ms) (Fukumoto et al., 2016; Kistler et al., 2004; Kojodjojo et al., 2006; Weber et al., 2011). These studies included a wide range of patients. The most striking result is the demonstration of an inverse correlation of CV with age (Kistler et al., 2004; Kojodjojo et al., 2006). In our study, there was no difference in age between the control patients and those with AF. While some studies have included patients with AF (Fukumoto et al., 2016; Weber et al., 2011), there is no single study comparing AF to controls using the same experimental technique. As such, our study comparing control patients to those with paroxysmal AF is unique. According to classic leading‐circle reentry, shorter WL is thought to increase the risk of reentry and thus AF (Nattel et al., 2017). It is interesting to note that WL was increased in our patients with AF (Figure 1c) compared with controls in both the right and LA. This observation is due to an enhanced CV (Figure 1b), and may be a feature of paroxysmal AF, but also suggests that other factors play an important role in the underlying pathophysiology of AF. For example, Na^+^‐channel‐blocking class I antiarrhythmic drugs suppress AF yet lead to reduced CV and WL (Suzuki et al., 2013). This observation is consistent with spiral wave rotors (Nattel et al., 2017) and is also consistent with our in vivo observations. Our patient population was also small (*n* = 4), and a larger study may identify a subpopulation of patients with AF (e.g., persistent AF) that have reduced CV and WL. Our finding that CV and ERP are independent of pacing frequency over the range 400–600 ms in consistent with an earlier report that short no dependent once the pacing frequency was >400 ms (Weber et al., 2011).

Our ex vivo measurements also involved a small number of donor hearts (*n* = 2). This may be the reason why we did not detect any differences between the LA and RA CV or WL. The differences in the longitudinal compared with transverse CV and WL highlight the spatial dependence in electrical conduction within the atrial that would be difficult to detect in vivo, but may play a role in the development of AF. Nonetheless, in addition to providing a useful benchmark for the hiSPC‐aCM‐derived in vitro model, the ex vivo model may also be a useful tool to advance our understanding of atrial conduction and AF.

In summary, utilizing relatively simple microfabrication technology, we patterned iPSC‐derived atrial cardiomyocytes into a linear 1D spiral pattern. This design facilitates direct measurement of WL by allowing visualization of the start of repolarization and the end of depolarization. The relatively narrow (200 μm) design significantly enhances CV compared with 2D planar patterning of the hiPSC‐aCM and matched the CV measured in human hearts both in vivo and in a novel ex vivo human atrial tissue slice. The hiPSC‐aCM‐derived 1D spiral model may be a useful tool in the patient‐specific management of diseases associated with atrial conduction such as AF.

## AUTHOR CONTRIBUTIONS

Sherri M. Biendarra‐Tiegs contributed to the experimental design, performed the experiments related to the 1D and 2D designs, analyzed the data, and contributed to writing the manuscript. Sergey Yechikov contributed to the experimental design, performed the experiments related to the creation of the hiPSC‐aCM, analyzed the data, and contributed to writing the manuscript. Bhupinder Shergill contributed to performing the 2D atrial conduction studies and writing the manuscript. Brittany Brumback contributed to the experimental design and collection of the ex vivo data and editing of the manuscript. Kentaro Takahashi contributed to the experimental design and collection of the ex vivo data, and editing of the manuscript. Venktesh S. Shirure contributed to the experimental design and data analysis. Ruth Estelle Gonzalez contributed to the experimental design and data analysis of the 1D design. Laura Houshmand contributed to the experimental design and performed the experiments related to the creation of the hiPSC‐aCM, and analyzed the data. Denise Zhong contributed to the experimental design and performed the experiments related to the creation of the hiPSC‐aCM, and analyzed the data. Kuo‐Chan Weng performed the patch‐clamp experiments and assisted in the data analysis. Jon Silva contributed to the experimental design and data analysis of the patch‐clamp experiments. Timothy W. Smith contributed to the experimental design, data collection, and analysis of the in vivo measurements. Stacey L. Rentschler assisted in the conception of the overall project, contributed to the experimental design, data analysis, and writing of the manuscript related to the in vivo and ex vivo measurements. Steven C. George conceived the overall project, contributed to the experimental design, data interpretation, and writing of the manuscript.

## FUNDING INFORMATION

This work was supported in part by grants from the National Institutes of Health (UH3 HL141800 [SCG and SAR], T32 HL086350 [SMB‐T], T32 HL125241‐03 [BB]) and the National Science Foundation (GRFP DGE‐1745038). Dr. Rentschler is the recipient of a Career Award for Medical Scientists from the Burroughs Wellcome Foundation.

## CONFLICT OF INTEREST

The authors do not have any conflict of interest.

## ETHICS STATEMENT

All procedures associated with the in vivo electrophysiological recordings were approved by the Institutional Review Board at Washington University in St. Louis, and written informed consent was obtained from each subject. Non‐failing human hearts were obtained from Mid‐America Transplant Services (MTS) in St. Louis, MO. Experimental protocols were approved by the Washington University in St. Louis Institutional Review Board (IRB). Informed consent was obtained for all tissue used in this study.

## Supporting information


Figure S1

Figure S2

Figure S3

Figure S4

Table S1
