# Supplementary material for: An iPS‐derived in vitro model of human atrial conduction
Source: Physiol Rep. 2022 Sep 18;10(18):e15407. doi: 10.14814/phy2.15407 (PMC9483613; doi:10.14814/phy2.15407)
Supplement: Supplementary file 5 — Table S1 [file PHY2-10-e15407-s002.pdf]

Table S1. qPCR primer sequences.

Gene name	Forward (5'-3')	Reverse (5'-3')
Pitx2C	ATGAACTGCATGAAAGGCCCGCT	TAAGGTTGGTCCACACAGCGATTT
Scn5a	CCAGATCTCTATGGCAATCCA	GAATCTTCACAGCCGCTCTC
Gapdh	GGCCTCCAAGGAGTAAGACC	AGGGGTCTACATGGCAACTG
Hey2	AAGGCGTCGGGATCGGATAA	AGAGCGTGTGCGTCAAAGTAG
Irx4	TTGGACTCCTGGGAACATGGACAA	ATGCTTCAGGGTATCTGGCCTCTT
Kcnj3	TCATCAAGATGTCCCAGCCCAAGA	CACCCGGAACATAAGCGTGAGTTT
Myl2	TGTCCCTACCTTGTCTGTTAGCCA	ATTGGAACATGGCCTCTGGATGGA
Myl7	ACATCATCACCCATGGAGACGAGA	GCAACAGAGTTTATTGAGGTGCCC
Nppa	GGGTCTCTGCTGCATTTGTGTCAT	AGAGGCGAGGAAGTCACCATCAAA
